# Voting for a personality: Do first impressions and self-evaluations affect voting decisions?

**DOI:** 10.1016/j.jrp.2014.04.011

**Published:** 2014-08

**Authors:** Markus Koppensteiner, Pia Stephan

**Affiliations:** Department of Anthropology/Human Behavior Research, University of Vienna, Austria

**Keywords:** Social judgments, Person perception, Similarity, Politics, Response surface analyses

## Abstract

Participants were asked to assess their own personality (i.e. Big Five scales), the personality of politicians shown in brief silent video clips, and the probability that they would vote for these politicians. Response surface analyses (RSA) revealed noteworthy effects of self-ratings and observer-ratings of openness, agreeableness, and emotional stability on voting probability. Furthermore, the participants perceived themselves as being more open, more agreeable, more emotionally stable, and more extraverted than the average politician. The study supports previous findings that first impressions affect decision making on important issues. Results also indicate that when only nonverbal information is available people prefer political candidates they perceive as having personality traits they value in themselves.

## Highlights

• People rated themselves and video clips of politicians on personality.
• People gave an estimate of the probability that they would vote for the politicians.
• Ratings of some personality traits were strongly related to voting behavior.
• For the preferred personality traits people gave themselves higher ratings.
• People’s voting decisions may be guided by traits they value high in themselves.

## 1 Introduction

People form first impressions on the basis of appearance and other nonverbal cues. Such cues not only elicit quick attributions of personality traits and emotional states but also appear to provide a sufficiently reliable source of information to support accurate assessment of personality (e.g. Ambady, Bernieri, & Richeson, 2000; Borkenau, Mauer, Riemann, Spinath, & Angleitner, 2004; Kenny, Horner, Kashy, & Chu, 1992). This ability might help to make social interaction smoother but it is undeniable that such snap judgments also affect public decision making. For instance, the verdicts of judges in small-claims courts have been shown to be at least somewhat influenced by the facial features of the defendant (Zebrowitz & McDonald, 1991). Similarly in the political arena: successful self-presenters create social bonds with an audience not only by finding the right words but also by displaying the “right” behavior (Cherulnik, Donley, Wiewel, & Miller, 2001; Stewart, Waller, & Schubert, 2009). Nonverbal cues can be so convincing that attributions of competence and other personality traits to photographs of political candidates have been successfully used to predict electoral outcomes (e.g. Antonakis & Dalgas, 2009; Ballew & Todorov, 2007; Olivola & Todorov, 2010; Poutvaara, Jordahl, & Berggren, 2009). Consequently, in addition to voter and candidate ideology (e.g. Caprara, Barbaranelli, Consiglio, Picconi, & Zimbardo, 2003; Roets & Van Hiel, 2009), politicians’ appearance and their nonverbal behaviors may also affect people’s voting behavior and how they judge candidate personality.

Communication, however, is not a one-way process. Although there is often no direct interaction between speakers on stage and their audience, information communicated has to be processed by the intended receivers. This processing depends in part on how the members of an audience perceive and relate to the speaker. Research has shown that people feel more closely connected to others they perceive to be similar to themselves in attitude and personality (e.g. Berscheid, 1966; Byrne, 1961; Tenney, Turkheimer, & Oltmanns, 2009). More pertinent to our research, this connection also appears to play a role in politics, particularly when people do not gather much information about candidates and their positions. For instance, people seem to vote for politicians whose personality traits are similar to their own (Caprara, Vecchione, Barbaranelli, & Fraley, 2007). Other findings even hint that the “similarity creates liking” relationship applies to physiognomic features. If voters are unfamiliar with politicians they seem to prefer candidates in whose faces they recognize themselves (Bailenson, Iyengar, Yee, & Collins, 2008).

The current study investigated the relationship between first impressions and people’s tendency to favor others they regard as having a similar personality. We conducted an experiment in which participants rated short video clips of politicians giving a speech. The main focus of the study was first impressions formed by nonverbal information; so to avoid interference from speech content and different degrees of prominence we presented silent video clips showing politicians that were unknown to our participants. Participants’ impressions were collected using a brief version of a Big Five personality inventory. We also asked participants to report their own personality and give an estimate of the likelihood that they would vote for the speakers they had seen.

Judging a stranger’s personality by brief displays of behavior may be fairly accurate (see above); however, people are often misled by first impressions and tend to simplify decision processes by relying on simple rules (e.g. Kahneman, 2003; Olivola & Todorov, 2010). Given that similarity creates liking, we assumed that people sometimes use their own personality as a kind of reference point when expressing preferences for others. This effect may be even more pronounced for personality traits people value highly and in situations of low information such as in our experimental setting. We used polynomial regression analyses with response surface plots to analyze the relationship between perceived personality, self-rated personality and voting probability (e.g. Edwards & Parry, 1993). These statistical procedures provide information about how congruence and incongruence between two independent variables relate to a dependent variable. They also yield coefficients describing the nature of the relationships between variables (i.e. linear and curvilinear), thereby providing a more comprehensive picture of how the different variables are interrelated than other statistical procedures.

In summary, on the basis of research providing evidence for “similarity effects” in different domains and the finding that people perceive themselves as being above average in ability and character (e.g. Alicke, Klotz, Breitenbecher, Yurak, & Vredenburg, 1995; Ehrlinger, Johnson, Banner, Dunning, & Kruger, 2008) we made the following predictions. First, we hypothesized that our participants tend to “vote for” politicians in which they perceive personality traits they also preferably ascribe to themselves. We did not have distinct hypotheses to which personality traits this applies but expected to find patterns of congruence between self-ratings and observer-ratings of some traits. To reveal such patterns of congruence we used the aforementioned response surface analyses (RSA), because this procedure provided detailed insights into whether and in what way self-ratings and observer-ratings are related to voting probability. Second, we assumed that potential candidates would be judged according to the self-attributed “high standards” of their voters. We therefore expected the participants of the rating experiment to perceive themselves as being above the average politician in those personality traits they preferably ascribe to themselves. In other words, for personality traits the participants valued highly we expected to find large differences between self-ratings and observer-ratings. Consequently, the aim of this study was not to show that people are able to make quite reliable guesses about someone else’s personality on the basis of brief displays of behavior, but that people integrate self-perceptions into the guesses they make.

## 2 Method

### 2.1 Participants

Eighty participants (42 females with age *M *= 23.1 years, *SD *= 3.7; 38 males with age *M *= 23.9 years, *SD *= 4.4) were approached personally at locations throughout the University of Vienna and asked to take part in a rating experiment. Raters were not reimbursed for participating in the experiment.

### 2.2 Stimulus preparation and procedure

We selected 40 speeches from the German houses of parliament (20 female and 20 male speakers), and randomly extracted brief video segments from each of the speeches to give 40 video clips with an average length of 15 s. The lower portion of the video clips was cut off to remove captions which gave information about the speaker’s name and party. The speakers we chose were ordinary, non-prominent members of the German Bundestag, who were not very well known in Germany and even less well-known in Austria. In addition, we asked participants if they recognized any of the speakers they had judged. None of the politicians were known to any participant.

Stimuli were presented using a rating program. The video clips were presented on the left-hand side of the program’s user interface; on the right-hand side 20 bipolar personality terms (e.g. outgoing and reserved) based on a brief German version of the NEO-FFI personality inventory (Borkenau & Ostendorf, 1991) were displayed. This questionnaire assesses the Big Five personality dimensions of extraversion, agreeableness, conscientiousness, neuroticism or emotional stability and openness. Below the list of personality items the user interface displayed an additional bipolar item: “I would vote for this candidate” or “I would not vote for this candidate”. Participants completed their ratings by dragging a “trackbar control” to the right or to the left pole of the bipolar scales using a computer mouse. The position of the bar corresponded to points along a semantic differential, which was divided into 100 subunits with 0 being the maximum value of the item on the left, 50 being the neutral position, and 100 being the maximum value of the item on the right. The same scale was used for the voting choice item, enabling participants to give an estimate of the probability that they would vote for a candidate. In order not to overtax the participants and to reduce the influence of stimulus order, each participant only rated a subset of eight speakers randomly selected from the 40 video clips.

### 2.3 Analysis

We aggregated the questionnaire data by summing items belonging to a latent personality dimension (i.e. each personality dimension comprised four items). We then averaged observer-ratings across the subsets of eight stimuli each participant had rated. The resulting dataset − consisting of 80 self-ratings, 80 mean observer-ratings (i.e. for each personality dimension), and 80 ratings on the continuous voting scale − was then analyzed by means of polynomial regressions and response surface analysis (RSA). Polynomial regression yields regression coefficients for two linear terms (i.e. observer-ratings and self-ratings in this study), their interaction (i.e. relationship between observer-ratings and self-ratings), and two quadratic terms (i.e. squares of observer-ratings and self-ratings) and relates them to an independent variable (i.e. voting probability). To guard against multicollinearity and to adjust differences in variances, which often affect the interpretation of multiple regressions coefficients, we applied *z*-standardization before the polynomial regression analyses.

The regression coefficients obtained by polynomial regression are used to calculate the response surface parameters *a*_1_–*a*_4_. These parameters define the response surface (RS) plane of a three dimensional plot. RS plots have, numerically, a line of congruence (LOC: *X = Y*) and a line of incongruence (LOIC: *X = −Y*), which are derived by fully crossing the numeric levels of two continuous predictor variables *X* and *Y*. The LOC is defined by a linear slope (*a*_1_) and a curvature (*a*_2_); similarly the LOIC is defined by a linear slope (*a*_3_) and a curvature (*a*_4_). Thus, the LOC and the LOIC provide insight into how congruence and incongruence between the independent variables are related to a dependent variable, which is plotted on the *Z*-axis (see Table 1 and for more information see Edwards & Parry, 1993; Schönbrodt, 2013; Shanock, Baran, Gentry, Pattison, & Heggestad, 2010).

**Fig. 1:**
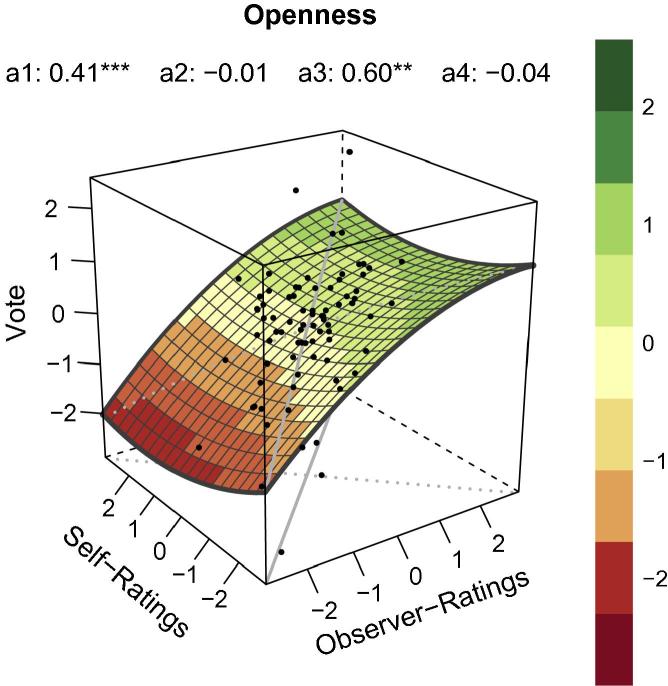
RSA plot for the personality dimension *Openness* based on polynomial regression analyses. Values were standardized via *z*-transformation. *X* = observer-ratings. *Y *= self-ratings. *Z *= vote = voting probability. Gray continuous line = line of congruence (LOC). Gray dotted line = line of incongruence (LOIC). Black dots = data points. *a*_1_ = linear relationship along LOC (*X = Y*). *a*_2_ = non-linear relationship along LOC. *a*_3_ = linear relationship along LOIC (*X = −Y*). *a*_4_ = non-linear relationship along LOIC.

**Fig. 2:**
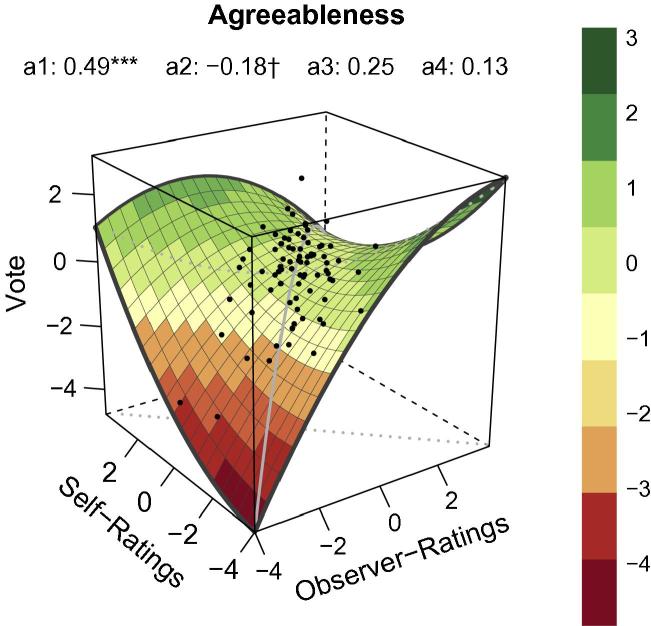
RSA plot for the personality dimension *Agreeableness* based on polynomial regression analyses. Values were standardized via *z*-transformation. *X *= observer-ratings. *Y *= self-ratings. *Z *= vote = voting probability. Gray continuous line = line of congruence (LOC). Gray dotted line = line of incongruence (LOIC). Black dots = data points. *a*_1_ = linear relationship along LOC (*X* = *Y*). *a*_2_ = non-linear relationship along LOC. *a*_3_ = linear relationship along LOIC (*X = −Y*). *a*_4_ = non-linear relationship along LOIC.

**Fig. 3:**
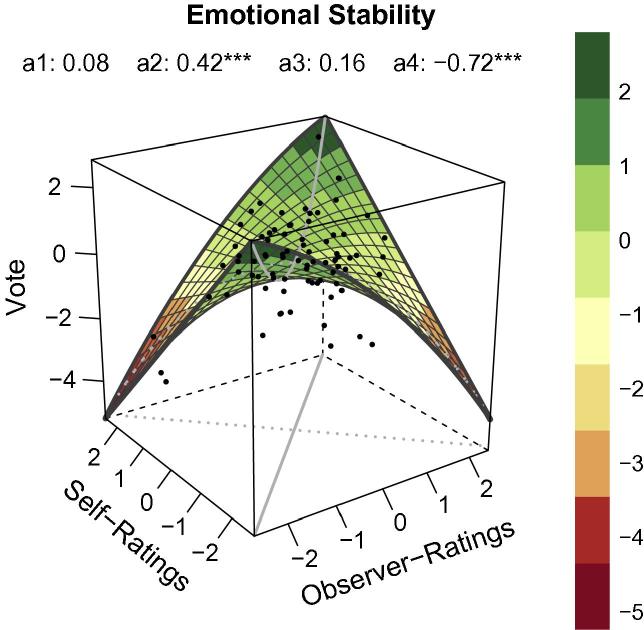
RSA plot for the personality dimension *Emotional Stability* based on polynomial regression analyses. Values were standardized via *z*-transformation. *X *= observer-ratings. *Y *= self-ratings. *Z *= vote = voting probability. Gray continuous line = line of congruence (LOC). Gray dotted line = line of incongruence (LOIC). Black dots = data points. *a*_1_ = linear relationship along LOC (*X = Y*). *a*_2_ = non-linear relationship along LOC. *a*_3_ = linear relationship along LOIC (*X = −Y*). *a*_4_ = non-linear relationship along LOIC.

Applied to our data this means that self-ratings and observer-ratings were plotted on the *X*-axis and the *Y*-axis of the RSA plot, while voting probability was plotted on the *Z*-axis (see Figs. 1–3). Thus, self-ratings and observer-ratings defined the plane of the three-dimensional plot. The LOC running from the near corner to the back corner of the plot was derived by crossing equal values of self-ratings and observer-ratings (i.e. where self-ratings of −1 corresponded with observer-ratings of −1; self-ratings of 0 corresponded with observer ratings of 0, etc.). The LOIC running from the left to the right corner of the plot was derived by crossing equal pairs of negative and positive values of observer-ratings and self-ratings (e.g. where −1 met +1). Along the LOC the parameter *a*_1_ estimated a linear additive effect (i.e. negative or positive slope) of self-ratings and observer-ratings on voting probability, whereas the parameter *a*_2_ gave information about the curvature (i.e. negative or positive) of this relationship. In contrast, the parameters *a*_3_ and *a*_4_ informed about how a linear or a non-linear relationship along the LOIC affected voting probability.

To examine which personality traits participants valued in themselves and how they perceived themselves in relation to the politicians they saw, we compared participants’ self-ratings with their average politician rating (i.e. the mean rating of eight politicians each participant rated). The results of these analyses are presented as *t*-tests with standard effect size measures (i.e. Cohen’s *d*), which in combination with the means give an estimate of the degree and direction in which self-ratings differed from observer-ratings.

All these statistical analyses were done using the statistical software package R (R Core Team, 2013).

### 2.4 Power analysis

We had no specific hypotheses about the degree to which the different dimensions of the Big Five traits would be related to voting probability. Other studies have found bivariate and multiple correlation coefficients for certain personality traits with voting behavior that explained more than 16% of total variance, so we expected that there would be a medium effect size for at least some dimensions (Olivola & Todorov, 2010; Poutvaara et al., 2009). We therefore assumed a medium effect (*R*^2^ = .15) for polynomial regression analyses with an alpha level of .05, a power level of .8, and five predictors. On the basis of these assumptions an a priori power analyses suggested an optimal sample size of 73 participants.

Our study was also exploratory in that regard that the *t*-tests and their corresponding effect size measures were used to determine in which personality traits self-ratings differed markedly from observer-ratings. We expected that for highly valued personality traits participants’ self-ratings would be at least moderately different from the average politician rating because other findings indicated that the better-than-average effect can be moderate to strong (Alicke et al., 1995; Ehrlinger et al., 2008). We therefore performed an a priori power analyses with a Cohen’s *d* of .5 (i.e. threshold for medium effect), an alpha level of .05, and a power of .8, which suggested an optimal sample size of 64 individuals for the *t*-tests we applied.

Power analysis was performed using the statistical software package R (Champley, 2012).

## 3 Results

On average each politician was rated by 16 different participants with a range of 13–19 ratings. Measures of the questionnaire’s internal consistency with regard to observer-ratings yielded moderate to high reliabilities for extraversion (Cronbach’s *α* = .80), agreeableness (*α* = .87), conscientiousness (*α* = .72), openness (*α* = .62), and emotional stability (*α* = .73). The participants’ self-ratings on the Big Five personality dimensions showed a similar pattern. There were moderate to high reliabilities for extraversion (*α* = .88), agreeableness (*α* = .84), conscientiousness (*α* = .82), and emotional stability (*α* = .70), but low reliability for openness (*α* = .50). For this reason, the interpretation of the results for openness presented below should be treated with some caution.

Results of response surface analyses (RSAs) are presented in Table 1. The proportion of the total variance explained ranged from *R*^2^ = .10 for extraversion to *R*^2^ = .37 for agreeableness. The parameters *a*_1_–*a*_4_ are central to RSA and provide estimates of congruence and incongruence. Therefore, interpretation of the results mainly focuses on these parameters. We assumed that similarity would play a role, but we had no specific hypotheses about which personality dimensions would show a self-observer similarity (or congruence) effect on voting behavior. In this regard our analysis was exploratory. Inspection of the correlation coefficients revealed that extraversion yielded the lowest *R*^2^, but also that none of the regression coefficients reached statistical significance. This indicates that there was a relatively weak relationship between self-ratings and observer-ratings for extraversion and voting probability. A similar interpretation can be applied to the data on conscientiousness. Although *R*^2^ for this personality trait was higher than for extraversion, none of the relevant RSA parameters were significant. In contrast, RSA for all the other Big Five personality dimensions yielded significant results for at least one of the relevant parameters. These results and the corresponding plots are discussed in greater detail in the following subsections.

### 3.1 RSA for openness

RSA for openness provided a relatively strong positive linear relationship (*a*_1_ in Table 1) along the LOC, which runs from the near corner to the far corner of the RSA plot (Fig. 1). At first sight this indicates a congruence effect and that voting probability increased as self- and observer-ratings of openness increased. However, the parameter *b*_1_ (i.e. observer-rating in Table 1) and *b*_2_ (i.e. self-rating in Table 1) of the polynomial regression revealed that observer-ratings of openness have a prevailing influence on the RSA parameter *a*_1_. Apparently the participants’ aptness to vote for somebody they perceived as open was predominantly affected by their first impressions. There was also a strong linear relationship (*a*_3_ in Table 1) along the LOIC, which runs from the left corner of the plot to its right corner indicating that self- and observer-ratings for openness showed both congruence and incongruence.

Inspection of Fig. 1 reveals more details how the different variables were related and supported interpretations based on the parameters of the polynomial regression. Overall, voting probability increased (i.e. higher values on *Z*-axis) when observer-ratings for openness increased. Low self-ratings combined with low observer-ratings (i.e. congruence) were not associated with a higher voting probability than high self-ratings combined with low observer-ratings. However, high observer-ratings were associated with higher voting probability regardless of whether they were combined with low self-ratings (i.e. incongruence) or high self-ratings (i.e. congruence). In conclusion, although data points in the RSA plot of openness appear to follow the LOC, we found no clear effect of congruence for openness. Irrespective of their self-rating, participants preferred politicians they rated highly for openness.

### 3.2 RSA for agreeableness

For agreeableness RSA indicated a strong linear relationship (*a*_1_ in Table 1) along the LOC. This suggests that congruence between self- and observer-ratings of agreeableness had an impact on voting probability. However, Fig. 2 shows that the relationship between self- and observer-ratings for agreeableness and voting probability were not so simple. When self-ratings and observer-ratings were low, voting probability was also low (i.e. low value on *Z*-axis of plot); concurrent increases in self- and observer-ratings were associated with higher voting probabilities (i.e. positive slope of *a*_1_). There was also a non-significant curvature (*a*_2_ in Table 1) along the LOC, indicating that voting probability decreased slightly when ratings for agreeableness were high. In addition, although RSA yielded no significant effect for incongruence (see *a*_3_ and *a*_4_ in Table 1), high voting probabilities were observed when agreeableness ratings were somewhat incongruent. For instance, some participants who rated themselves high on agreeableness also tended to choose higher voting probabilities even for politicians they rated not agreeable.

In summary, conclusions for agreeableness resembled the conclusions we drew for openness. Although congruence effects were more pronounced for agreeableness than for openness, RSA indicates that participants mostly based their judgments on first impressions and preferred politicians they perceived as being highly agreeable. In comparison with this self-ratings of agreeableness seemed to be of minor importance. Consequently, we found no clear similarity effect for this personality trait but a strong effect of perceived agreeableness on voting probability.

### 3.3 RSA for emotional stability

RSA for emotional stability yielded a pronounced positive curvature along the LOC (*a*_2_ in Table 1) and a pronounced negative curvature along the LOIC (*a*_4_ in Table 1). Consequently, self-ratings and observer-ratings of emotional stability produced non-linear effects of congruence and incongruence. The significant negative slope of parameter *a*_4_ (i.e. producing a concave curvature along the LOIC) enhanced the robustness of the congruence effect along the LOC. It shows that voting probability decreased when combinations of self-ratings and observer-ratings were “far from the LOC” and incongruence between these variables was strong.

Inspection of Fig. 3 provides more clarity. The path of the LOC in Fig. 3 reveals that voting probability was very high when low ratings were given for both own emotional stability and speakers’ emotional stability. Voting probability decreased slightly at moderate levels of congruence, and rose again at higher levels of congruence between self-ratings and observer-ratings for emotional stability. Slight incongruence was also associated with high voting probability but when self-ratings and observer-ratings for emotional stability differed too much (i.e. low self-ratings combined with high observer-ratings and vice versa), voting probability was lower. Overall, in comparison with the other personality traits results for emotional stability provided the clearest congruence effect.

### 3.4 Differences between self-ratings and observer-ratings

The comparison of observer-ratings of personality with self-ratings revealed that on average participants gave themselves higher ratings on all five personality dimensions (see Table 2). Exploratory analyses of these differences with *t*-tests and effect size measures revealed strong effects for openness, agreeableness and emotional stability. A medium effect size was found for extraversion and a very low one for conscientiousness. This shows that participants believed themselves to be far more open, agreeable and emotionally stable than the average politician. However, effect size measures (Cohen’s *d*) should be interpreted with some caution because there were violations of variance homogeneity.

## 4 Discussion

In this study we asked participants to rate themselves and short video clips of politicians on scales measuring the Big Five personality traits and to give an estimate of the probability that they would vote for each politician they evaluated.

We used polynomial regression and response surface analyses to examine whether the participants were more likely to vote for politicians they perceived to be similar to themselves in terms of personality. We expected similarity to have an effect, but we had no specific hypotheses about which personality dimensions played a role. It was revealed that regression models for openness, agreeableness, and emotional stability explained a high proportion of the variance in voting probability. Less variance was explained by models for extraversion and conscientiousness. This suggests that our student sample regarded extraversion and conscientiousness as personality traits of minor importance to voting decisions. A tendency to assign a higher voting probability to speakers that were perceived to be agreeable is perhaps not surprising as politicians should appear likeable. However, the propensity shown by our sample to attach great importance to openness and minor importance to conscientiousness when making voting decisions might only hold true for a student sample such as the one used in our experiment. Future studies should use samples from different social groups, because it has been shown that the relationships between political attitude and personality traits depend on social context and ideology (e.g. Gerber, Huber, Doherty, Dowling, & Shang, 2010; Roets & Van Hiel, 2009) and it is reasonable to assume that this also extends to perceptions of politicians’ personalities.

Although we found some incongruence between rater-politician similarity and its relationship to voting probability, detailed analyses showed that participants tended to vote for “someone like me” when the candidate was perceived to have similar emotional stability. There were also similarity effects for agreeableness and openness but these were less clear; the most prominent difference from the similarity effect observed for emotional stability was that for openness and agreeableness concurrent low self- and observer-ratings were not associated with higher voting probability. In addition, in both cases observer-ratings were the more dominant contributor to the found similarity effects and had a prominent effect on voting probability. It is conceivable that observer-ratings on these personality traits have to reach a certain level before a similarity effect delivers an increase in voting probability. People refrain from voting for a candidate they rate as low on the personality traits they favor in political candidates. Such reasoning may also be applied to emotional stability, but the surprising result was that for this personality trait voting probability was highest when low self-ratings were paired with low observer-ratings or high self-ratings were paired with high observer-ratings. In between, at moderate levels of self- and observer-ratings voting probability was less high, although the effect was still strong.

Explanations for this pattern of results are speculative at present. Our measure of emotional stability contained items such as “aroused” vs. “composed”, which are adjectives that are typical of temporary emotional states occurring during an experiment. It is possible that participants matched their current emotional states with those they ascribed to the speakers they saw. Some participants may have preferred a highly aroused politician because they were also easily aroused; others may have preferred a very composed politician because they were very composed. This kind of matching behavior might have influenced voting decisions more strongly at the extremes of emotional stability.

Student’s *t*-tests revealed that in nearly all cases the participants gave themselves higher ratings for the traits they seemed to value highly in politicians they would vote for (see previous section). Participants regarded themselves as being more open, more agreeable, more emotionally stable and more extraverted than the average politician in our sample. Whilst this might be due to the poor public image of politicians, it is common to find that people regard themselves as “better than average” (e.g. Ehrlinger et al., 2008).

Surprisingly, no noteworthy difference between self-ratings and observer-ratings could be found for conscientiousness, which suggests that our student sample did not value conscientiousness as highly as the other personality traits we investigated. This result may be due to the difficulty of evaluating conscientiousness from brief displays of behavior. However, taking into account the results of the regression analyses and findings suggesting that the easy-to-recognize personality trait of extraversion (e.g. Kenny et al., 1992) was not particularly highly valued, it is plausible to suggest that people want their politicians to reach their own “high standards” on valued traits. Such a conclusion might be applied to openness and agreeableness in this study. As decision making is not confined to the political domain, the current findings may also be interpreted in a broader context. It is possible that in first encounters, during which a common problem has to be solved, people favor others they rate as closer to their self-rated “high standard” on certain personality traits.

In contrast to other studies which have found that voters preferred politicians with similar personality traits (Caprara et al., 2007) our study was based on appearance cues and first impressions. This may more closely resemble the situation in which politicians find themselves when they are newcomers to the political arena or when they are candidates for a new party about which ideological information is scarce. Furthermore, research suggests that even when more information is available voting decisions are often guided by superficialities and nonverbal cues (e.g. Antonakis & Dalgas, 2009; Olivola & Todorov, 2010).

Our experimental set-up has limitations, because we removed speech content and party membership from the video clips. Therefore, caution in extrapolating the results to real life situations is advised and additional work needed to underpin the findings. However, in follow up studies other potentially influential variables can be easily added to the experimental design and tested under controlled conditions. Manipulating the experimental setting this way could bridge the gap between studies that examine the role of nonverbal cues and those that examine the role of ideology in decision making and help to disentangle the role of appearance from that played by other sources of information. Inclusion of variables on party membership, for instance, might reveal whether there is some kind of an “attraction leads to similarity” effect when people make their voting decisions (e.g. Morry, 2007). It is conceivable that people who receive more background information “adapt” their ratings of politicians to make them more or less similar to themselves. In addition, future experiments should enable both randomization of the presented stimuli and assignment of same sets of stimuli to different participants. This would provide insights into inter-judge agreement and to what degree it varies for ratings of different personality dimensions.

In summary, our study supports previous findings showing that first impressions and visual appearance cues affect public decision making processes (see also Antonakis and Dalgas, 2009; Ballew & Todorov, 2007; Poutvaara et al., 2009). However, we extended earlier research by investigating the relationship between self-ratings, observer-ratings and voting probability with response surface analyses (RSA). This statistical tool enabled us to uncover relationships among the investigated variables that traditional methods of analyses would not have revealed. For instance, we found similarity effects for agreeableness, emotional stability and openness, but closer examination of the RSA plots showed that these effects had different appearances. RSA might thus help to refine hypotheses about similarity effects for personality traits by providing a more detailed picture of relationships than other analytical tools. All in all, our results suggested that people want a candidate to possess personality traits they believe themselves to possess and which therefore have a very high value for them.

## Figures and Tables

**Table 1:** Polynomial regression analyses of self-ratings and observer-ratings of politicians’ personality with voting behavior.

	Personality dimension				
	Openness	Conscientiousness	Agreeableness	Emotional stability	Extraversion
*Polynomial regression findings*					
*b*_1_ Observer-rating (SE)	.51 (.12)⁎⁎⁎	.04 (.12)	.37 (.12)⁎⁎	.12 (.10)	−.17 (.14)
*b*_2_ Self-rating (SE)	−.10 (.11)	.06 (.11)	.12 (.11)	−.04 (.09)	−.02 (.12)
*b*_3_ Observer-rating^2^ (SE)	−.08 (.10)	.01 (.12)	−.11 (.06)	−.16 (.06)⁎	.02 (.14)
*b*_4_ Observer-rating x self-rating (SE)	.01 (.16)	.24 (.13)	−.16 (.11)	.57 (.10)⁎⁎⁎	.17 (.15)
*b*_5_ Self-rating^2^ (SE)	.05 (.10)	−.21 (.10)⁎	.08 (.08)	.00 (.09)	.17 (.09)
*R*^2^	.21⁎⁎	.16⁎	.37⁎⁎⁎	.34⁎⁎⁎	.10

*Response surface parameters*					
*a*_1_ (SE)	.41 (0.12)⁎⁎⁎	.10 (.16)	.49 (.13)⁎⁎⁎	0.08 (.12)	−.18 (.18)
*a*_2_ (SE)	−.01 (.10)	.04 (.15)	−.18 (.11)	.42 (.11)⁎⁎⁎	.36 (.24)
*a*_3_ (SE)	.60 (.20)⁎⁎	−.01 (.17)	.25 (.18)	.16 (.15)	−.15 (.19)
*a*_4_ (SE)	−.04 (.30)	−.45 (.26)	.13 (.18)	−.72 (.17)⁎⁎⁎	.03 (.19)

*Notes*: *df*_1_ = 5, *df*_2_ = 74; *SE = *Standard error (reported in parentheses). Polynomial regression coefficients (*b*_1_ − *b*_5_) are standardized *β*-weights. RSA parameters are calculated as follows: *a*_1_ = *b*_1_ + *b*_2_; *a*_2_ = *b*_3_ + *b*_4_ + *b*_5_; *a*_3_ = *b*_1_ − *b*_2_*; a*_4_ *= b*_3_ − *b*_4_ *+ b*_5_*.*

⁎ *p* ⩽ .05.

⁎⁎ *p* ⩽ .01.

⁎⁎⁎ *p* ⩽ .001.

**Table 2:** Descriptive statistics and comparison of self-rated personality and ratings of politicians’ personality by Student-*t*-test.

	Personality dimension				
	Openness	Conscientiousness	Agreeableness	Emotional stability	Extraversion
*M* (observer-ratings)	214.51	239.75	206.35	206.28	217.54
*SD* (observer-ratings)	22.63	28.13	27.47	27.61	26.49
*M* (self-ratings)	264.04	241.73	298.29	246.36	241.48
*SD* (self-ratings)	44.41	72.01	58.66	58.21	76.06
*t*	8.89⁎⁎⁎	.23	12.70⁎⁎⁎	5.57⁎⁎⁎	2.66⁎⁎
*df*	117.42	102.56	112.07	112.84	97.89
Cohen’s *d*	1.41	.04	2.02	.89	.42
*SEM*	5.58	8.64	7.24	7.21	9.01

*Notes*: *SEM* = Standard error of the mean. *df* = 158. *df*s for *t*-tests have been corrected due to violations of variance homogeneity. Cohen’s *d* = standardized effect size measure for mean differences (*d* = 2*t*/√*df*). ^⁎^ *p* ⩽ .05.

⁎⁎ *p* ⩽ .01.

⁎⁎⁎ *p* ⩽ .001.
